# Genetic and Transcriptome Analyses of Callus Browning in Chaling Common Wild Rice (*Oryza rufipogon* Griff.)

**DOI:** 10.3390/genes14122138

**Published:** 2023-11-27

**Authors:** Lingyi Qiu, Jingjing Su, Yongcai Fu, Kun Zhang

**Affiliations:** National Center for Evaluation of Agricultural Wild Plants (Rice), Department of Plant Genetics and Breeding, China Agricultural University, Beijing 100193, China; 18310251347@163.com (L.Q.); 15837115546@163.com (J.S.); yongcaifu@cau.edu.cn (Y.F.)

**Keywords:** Chaling common wild rice, culturability, QTL, callus browning, WGCNA

## Abstract

Callus browning during tissue culture of *indica* rice is genotype dependent, thus limiting the application of genetic transformation for editing-assisted breeding and elucidation of gene function. Here, using 124 introgression lines (HCLs) derived from a cross between the *indica* rice 9311 and Chaling common wild rice and 2059 SNPs for single-point and interval analysis, we identified two major QTLs, *qCBT7* on chromosome 7 and *qCBT10* on chromosome 10, related to callus browning, explaining 8–13% of callus browning. Moreover, we performed RNA-seq of two introgression lines with low callus browning, HCL183 and HCL232, with *Oryza*. *rufipogon* introgression fragments on chromosomes 10 and 7, respectively. Three candidate genes (Os07g0620700, Os10g0361000, and Os10g0456800) with upregulation were identified by combining interval mapping and weighted gene coexpression network analysis using the DEGs. The qRT-PCR results of the three candidate genes were consistent with those of RNA-seq. The differentiation of *indica* and *japonica* subspecies *Oryza*. *sativa* and *Oryza*. *rufipogon* suggests that these candidate genes are possibly unique in *Oryza*. *rufipogon*. GO analyses of hub genes revealed that callus browning may be mainly associated with ethylene and hormone signaling pathways. The results lay a foundation for future cloning of *qCBT7* or *qCBT10* and will improve genetic transformation efficiency in rice.

## 1. Introduction

Plant genetic transformation is an important tool for gene functional research as well as genome editing and crop breeding and has enabled fundamental discoveries in plant biology and revolutionized commercial agriculture [1]. Efficient in vitro callus generation is needed for developing in vitro callus systems [2]. The initial step of Agrobacterium (*Agrobacterium tumefaciens*)-mediated plant transformation is in vitro plant tissue culture. However, compared to *japonica* (*Oryza sativa* ssp. *japonica*), cultivated rice (*O. sativa*) ssp. *indica* is recalcitrant to genetic transformation; in particular, the browning that occurs during callus proliferation is a major bottleneck in transforming *indica* accessions [3,4]. Callus browning leads to reduction in regenerative ability, growth retardation, and cell death [2,3]. Callus browning is highly genotype dependent in most *indica* varieties [3,5]. In addition, with the acceleration of industrialization of genetically modified crops, the safety of genetically modified organisms has attracted much attention. How to improve the safety of genetically modified organisms is an important issue we are facing. Plant phosphomannose isomerases have been successfully transformed into rice as a safe selectable marker gene [6]. However, the most-used selection systems have safety risks [7,8,9]. Therefore, on the one hand, the selected materials with light callus browning can provide good acceptor materials for genetic transformation; on the other hand, mining key culturability genes that are safe, efficient, and have independent intellectual property rights should improve culturability and genetic transformation efficiency.

Quantitative trait loci (QTLs) associated with culturability (including callus induction, proliferation, and regeneration) have been obtained in rice [5,10,11,12,13,14,15,16,17,18]. Furthermore, many studies have identified candidate genes affecting somatic embryogenesis in different plant species. Examples of such genes include *somatic embryogenesis receptor-like kinases* (*SERKs*) from potted rose (*Rosa hybrida* cv. Linda) [19]; *LEAFY COTYLEDON1* (*LEC1*) [20], *LEC2* [21], *BABY BOOM* (*BBM*) [22], and *WUSCHEL* (*WUS*) in cotton (*Gossypium hirsutum*) and Arabidopsis (*Arabidopsis thaliana*) [23]; and *WUSCHEL-related homeobox5* (*WOX5*) [24], *DNA binding with one finger5.6* (*DOF5.6*), and *DOF3.4* in wheat (*Triticum aestivum*) [25]. In addition, the causal genes behind two QTLs related to culturability have been identified in rice via map-based cloning. The main gene for one of these QTLs, *NiR,* which regulates regeneration ability, encodes a ferredoxin–nitrite reductase. *NiR* in *indica* cultivar Kasalath was introduced alone into the recalcitrant *japonica* cultivar, which typically exhibits callus browning during tissue culture. Notably, the expression of *NiR* has been offered as an easy selection marker for transformation by preventing callus browning and allowing callus to regenerate into entire transgenic plants [11]. Our previous study showed that a common wild-rice-derived *BROWNING OF CALLUS1* (*BOC1*) allele on chromosome 3 is responsible for the induction of callus browning and genetic transformation efficiency [3]. Until now, however, few genes controlling callus browning have been cloned, and the underlying regulatory networks remain unclear.

The regulatory mechanisms involved in callus browning have been widely investigated. The most studied mechanisms are physiological and biochemical indicators, such as polyphenol oxidase (PPO) [4,26], antioxidant enzyme activity [27], and nitrite reductase activity [28]. Plant hormones like ethylene [29,30] can aggravate callus browning. In our previous study, we suggested that callus browning might be mitigated by inhibiting cell senescence and cell death [3]. Deciphering the regulatory pathway of callus browning remains pertinent.

Transcriptome deep sequencing (RNA-seq) is a powerful tool for understanding the genes that respond to callus browning [3]. Weighted gene coexpression network analysis (WGCNA) facilitates the construction of regulatory networks and the identification of hub genes for target traits [31]. Many hub genes involved in maize (*Zea mays*) callus induction have been identified by combining genome-wide association studies (GWAS) and WGCNA [31]. However, no major QTLs regulating callus browning in rice have been identified by linkage analysis combined with WGCNA, and no potential regulatory network associated with callus browning has been analyzed using WGCNA.

The wild species germplasm of rice is crucial for rice improvement as it provides valuable genetic resources. Here, using linkage analysis, we identified several QTLs involved in callus browning from a set of introgression lines (named HCLs) derived from Chaling common wild rice (CLCWR) with cultivar 9311 as the recurrent parent. We also performed RNA-seq using two introgression lines, HCL183 and HCL232, exhibiting low callus browning and containing introgressed fragments on chromosomes 7 and 10 overlapping with the above QTL intervals. By combining differential gene expression data and WGCNA, we identified several hub genes and explored the possible regulatory pathways involved in callus browning. Our findings provide a good receptor material for genetic transformation; important gene resources for improving culturability and genetic transformation efficiency; and a basic molecular framework for callus browning, which will further lay the foundation for the development of selectable markers that are safe, efficient, and have independent intellectual property.

## 2. Results

### 2.1. Callus Browning Phenotypes in the Introgression Lines and the Recurrent Parent 9311

**Figure 1:**
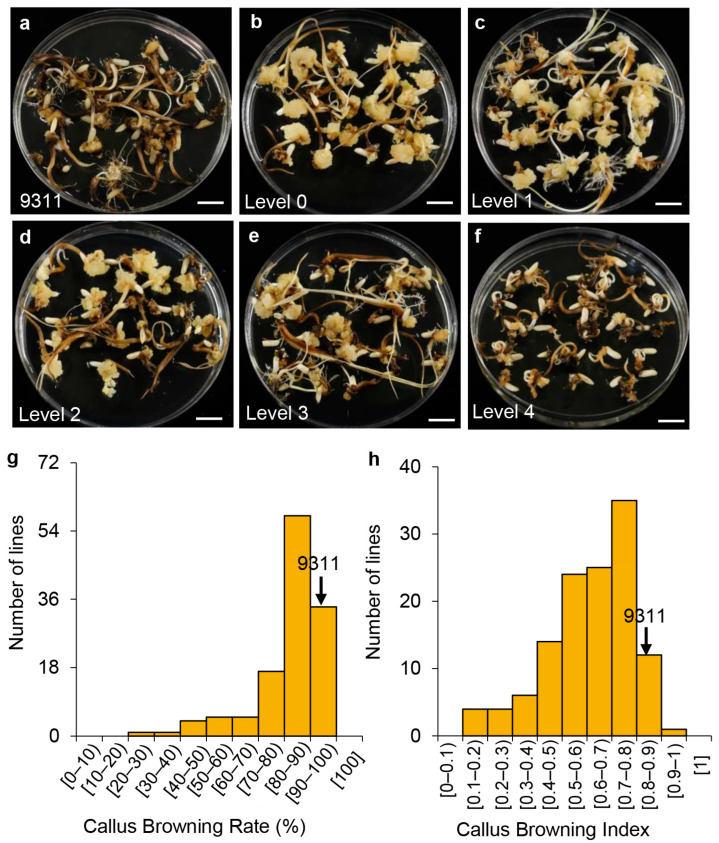
The phenotypes in the introgression lines and the rice cultivar 9311. (**a**–**f**) The phenotypes of 9311 and various rice introgression lines (HCLs) ranging from level 0 to 4 based on the scoring system used in our previous study [3]. Scale bars, 1 cm. (**g**,**h**) Frequency distribution of the callus browning rate (CBR) and callus browning index (CBI) in the HCLs. The values for 9311 are indicated by black arrows.

Our preliminary study showed that the callus browning index (CBI) of Chaling common wild rice (CLCWR, *Oryza rufipogon* Griff.) was 0.29, indicating that this accession is resistant to browning as fully susceptible accessions would have a CBI close to 1 [3]. To evaluate the genetic basis of the callus browning phenotype, we selected 124 introgression lines (HCLs) as the donor derived from recurrent backcrosses using CLCWR and the elite *indica* cultivar 9311, which is susceptible to browning, as the recurrent parent (Figure 1a–f). We used two indices to evaluate callus browning: the callus browning rate (CBR) and the CBI. The CBR and CBI of 9311 were 94.3% and 0.8, respectively, indicating high susceptibility to callus browning (Figure 1g,h). The CBR and CBI of the 124 HCLs were 37.9–100% and 0.1–1.0, respectively (Figure 1g,h). We detected significant differences in the variance between the HCLs (Table 1). Furthermore, we observed a positive correlation between CBR and CBI (Table 2). These results indicate that an analysis for QTLs reflecting callus browning potential can be performed using this set of introgression lines.

### 2.2. QTL Analysis of CBR and CBI

**Figure 2:**
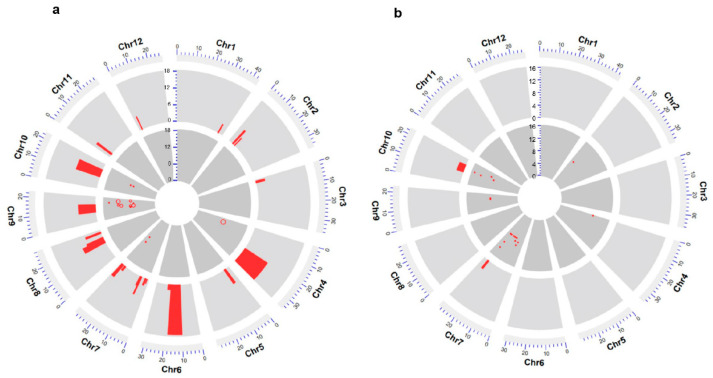
QTL distributions of CBR (**a**) and CBI (**b**). The first layer is the result of interval mapping, and the second layer is the result of single-point mapping. Each red box represents a QTL; the width of the red box represents the interval of the QTL, and the height represents the LOD value. The red dot represents the phenotypic variation.

We previously carried out the genotyping of 124 HCLs using a 1 K single nucleotide polymorphism (SNP) genotyping chip, which identified 2059 SNPs. Using CBR and CBI values as phenotypes, we performed QTL analysis using single-point and interval mapping methods. The single-point analysis identified 14 QTLs related to CBR and 18 QTLs associated with CBI. Importantly, the additive effects of QTLs responsible for CBR and CBI were negative, indicating that the alleles derived from CLCWR decrease browning incidence (Figure 2a,b; Table 3). In particular, the QTLs *qBR4-1*, *qBR7-1*, *qBR9-1*, *qBR9-2*, *qBR9-3*, *qBR9-4*, and *qBR10-1* on chromosomes 4, 7, 9, and 10 (as indicated in the QTL name) explained 10%, 10%, 16%, 13%, 13%, 12%, and 10% of the phenotypic variation, respectively (Figure 2a; Table 3). The QTLs *qBI10-1*, *qBI10-2*, *qBI10-3*, *qBI7-1*, *qBI7-2*, *qBI7-3*, and *qBI4-1* explained 15%, 13%, 10%, 13%, 11%, 10%, and 11% of the low CBI values seen in CLCWR, respectively (Figure 2b; Table 3). The interval mapping analysis for CBR showed that the QTLs were located on all 12 chromosomes (Figure 2a; Table 4); however, the QTLs for CBI were located within the region of 24,092,079 to 25,681,731 bp on chromosome 7 (named *qCBT7*) and 10,588,708 to 17,676,090 bp on chromosome 10 (named *qCBT10*) (Figure 2b; Table 4). Together, *qCBT7* and *qCBT10* were detected in QTL analysis of CBR and CBI, which proved that these two loci were real and credible.

### 2.3. Identification of Coexpression Network and Hub Genes

**Figure 3:**
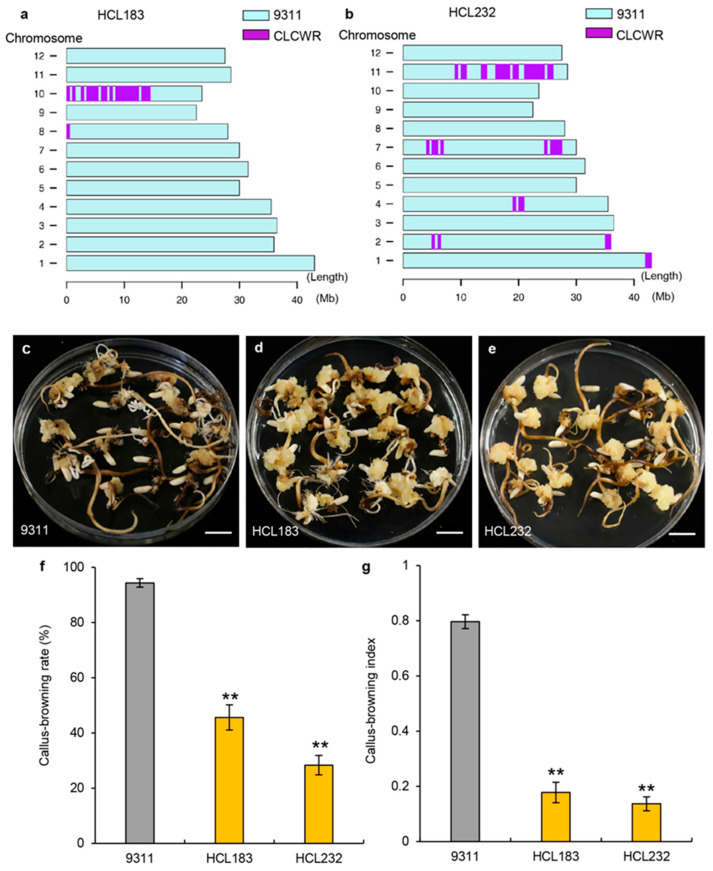
Callus browning phenotypes of introgression lines HCL183 and HCL232 and the recurrent parent 9311. (**a**,**b**) Genomic location of the introgressed fragments in HCL183 and HCL232. (**c**–**e**) Callus browning phenotypes of 9311, HCL183, and HCL232. Scale bars, 1 cm. (**f**,**g**) CBR (**f**) and CBI (**g**) in 9311, HCL183, and HCL232. The gray columns represent CBR and CBI of 9311 and the yellow columns represent these of HCL183 and HCL232. Data are presented as means ± standard deviation (SD, *n* = 3). **, *p* < 0.01; significant difference determined by the Student’s *t*-test.

**Figure 4:**
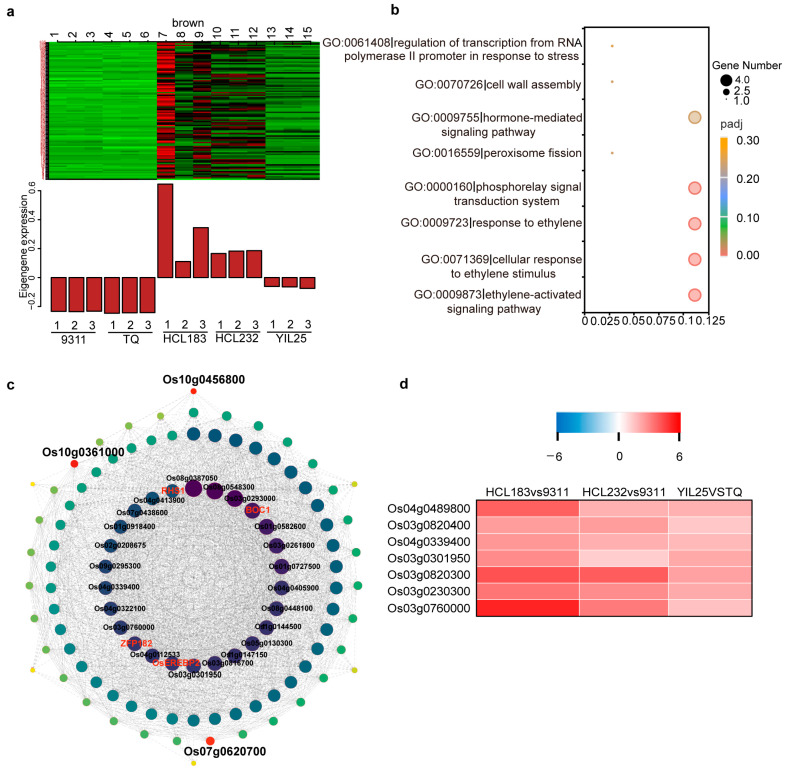
Coexpression network regulating callus browning. (**a**) The eigengene expression of the brown module, which is assigned to highly correlated stages, is shown in the figure. The expression pattern of module feature values in different samples is depicted. (**b**) The significantly enriched GO terms for genes in the brown module are presented. The figure shows the ratio of DEG numbers annotated to a specific GO term to the total number of DEGs. *X*-axis represents this ratio, and the size of each point corresponds to the number of genes annotated to the GO term. The color gradient from pink to orange indicates the significance of enrichment. (**c**) The coexpression network of hub genes is displayed. Gene connectivity selects a soft threshold with weights ranging from 0.25 to 1. The size and color of the circles represent the importance of the gene, with larger and darker nodes indicating higher importance in the network. Three candidate genes are highlighted in red, and the names of the cloned hub genes are shown in red font. (**d**) The heatmap of hub genes is presented. The table is color coded to show expression differences between different breed combinations according to the corresponding color legend.

To identify candidate genes associated with callus browning, we screened two introgression lines, HCL183 and HCL232, with light-browning phenotypes. HCL183 carried introgressed fragments from CLCWR on chromosome 10 (Figure 3a), while HCL232 harbored introgressed fragments from CLCWR on chromosome 7 (Figure 3b). The CBR and CBI values of HCL183 and HCL232 were significantly lower than those of 9311 (Figure 3c–g). We performed RNA-seq analysis of calli from 9311, HCL183, and HCL232 that had been induced on a callus-induction medium in the dark for 30 days. We identified differentially expressed genes (DEGs) between 9311 and either HCL. We also downloaded the list of DEGs between the *indica* cultivar TQ with high callus browning and the introgression line YIL25 with low callus browning, derived from a cross between the Yuanjiang common wild rice (*O. rufipogon* accession, YJCWR) with light callus browning and TQ from our previous publication [3] (Figure S1a–c). Compared to TQ, the CBR and CBI were lower in YIL25 (Figure S1d,e). Combining the DEGs between YIL25 and TQ and those identified between HCL183 and 9311 or HCL232 and 9311, we identified a total of 427 DEGs, including 149 upregulated and 278 downregulated ones. The 427 DEGs were classified into different coexpression modules. Module eigengenes were used to calculate the correlation coefficient with the CBI. The results indicated that deeper color represented a higher correlation (Figure S2a,b). WGCNA divided these 427 DEGs into three modules whose overall expression levels were also highly correlated with the CBI phenotypic data (Figure S2a,b). We confirmed the brown module to be a core module as the overall expression level of this module was highly and negatively correlated with the browning phenotypes (Figure 4a). Through gene enrichment and functional annotation analysis, we found that the genes in the brown module were enriched in pathways such as ‘ethylene-activated signaling’, ‘phytohormone-mediated signaling’, ‘cell wall assembly’, and ‘peroxisome’ (Figure 4b). K-within represents the connectivity of each gene within a single module to all other genes within the same module. A higher value of k-within indicates a higher level of connectivity for the gene, making it more likely to be the core gene of the module. We then sorted the genes in the brown module based on their k-within values and selected the top 20% hub genes. Network visualization of the hub genes was performed using the Omicshare platform (Om-icshare, http://www.omicshare.com/tools/, accessed on 29 August 2023) (Figure 4c). We identified several hub genes that may be related to the differences in callus browning phenotypes between the five materials used. For example, *BOC1*, which we had previously cloned, decreases callus browning by lowering oxidative-stress-induced cell senescence and cell death [3]; the transcription factor gene *OsEREBP2* is involved in salt stress in rice and may be essential in regulating responses to different abiotic stresses [32]; and TFIIIA-type zinc finger gene *ZFP182* participates in plant development and abiotic stress by participating in ABA-induced antioxidant defense process [33]. *RICE HIGH SHATTERING 1* (*RHS1*) is a negative regulator of rice seed shattering that modulates the redox signaling molecule S-nitrosothiol [34] and other genes like cytochrome P450 genes (Os03g0760000), *PEROXISOME BIOGENESIS FACTOR 11* (*PEX11*, Os03g0301950), and oxidoreductase (Os04g0339400). We have displayed the expression of these hub genes that may be related to callus browning in the form of a heat map in Figure 4d.

### 2.4. Candidate Gene Analysis with Significant Loci in the Intervals Involved in Callus Browning

**Figure 5:**
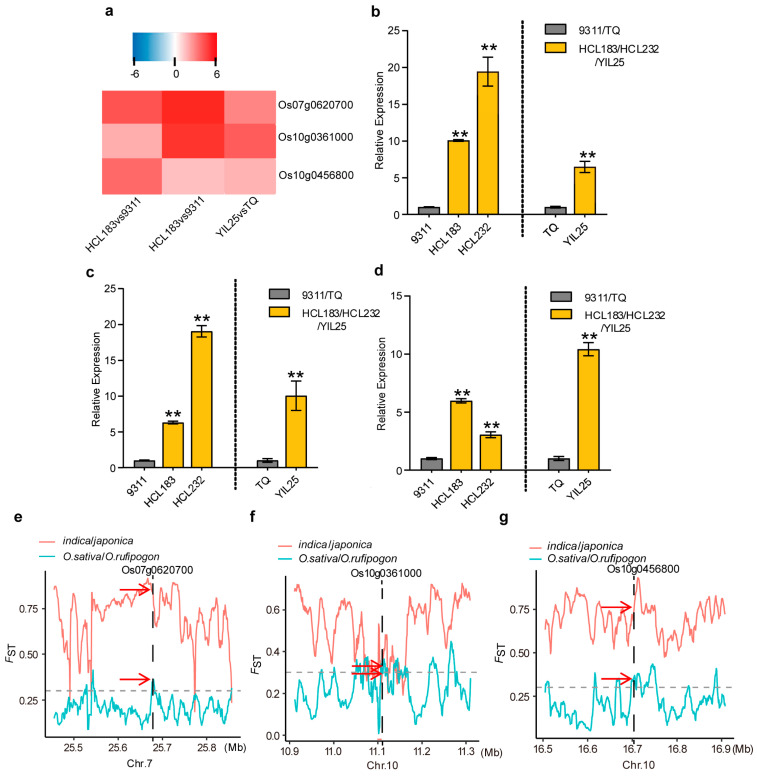
The relative expression and domestication analysis of candidate genes. (**a**) Heatmap representation of the log_2_foldchange of the three candidate genes. (**b**–**d**) The relative expressions of Os07g0620700, Os10g0361000, and Os10g0456800. All values are means ± SD (*n* = 3). **, *p* < 0.01, two-tailed test. (**e**–**g**) Fixation index (*F*_ST_) on chromosomes 7 and 10 between the *indica* and *japonica* subspecies *O. sativa* and *O. rufipogon*. The position where the black dotted line overlaps with the broken line (the red arrow) is the *F*_ST_ value of the three candidate genes. The dashed gray line represents a critical value of 0.3.

Combining the differentially expressed genes between YIL25 vs. TQ and WGCNA analysis, we identified three significantly upregulated candidate genes within the significant loci involved in callus browning on chromosomes 7 and 10 (Figure 5a). Os07g0620700, encoding a conserved hypothetical protein, was present in the candidate region for *qCBT7.* We also detected two genes within the candidate region of *qCBT10*. Os10g0361000, encoding a dehydration stress-induced protein, plays an important regulatory role in alleviating drought stress, protects cell membrane stability, decreases oxidative damage, and improves plant abiotic stresses tolerance to low temperature, drought, and salt [35]. *DST CO-ACTIVATOR 1* (*DCA1,* Os10g0456800, Figure 5a), encoding a CHY-type zinc finger protein acting as the transcriptional coactivator of *DROUGHT AND SALT TOLERANCE* (*DST*), regulates the expression of genes related to reactive oxygen species homeostasis [36]. Furthermore, the qRT-PCR results were consistent with those of RNA-seq in the expression levels of the three candidate genes, and the expression was remarkably increased in the low callus browning introgression lines HCL183, HCL232, and YIL25 (Figure 5b–d). Meanwhile, using publicly available genome resequencing data containing 446 accessions of *O. rufipogon* and 1083 *O. sativa* on a sliding window [37], we analyzed the fixation index (*F*_ST_, the level of population differentiation) of the three candidate genes. The *F*_ST_ level in this candidate interval was greater than 0.3 between the *indica* and *japonica* subspecies *O. sativa* and *O. rufipogon* (Figure 5e–g). In particular, the *F*_ST_ of Os07g0620700 was as high as 0.8, and the *F*_ST_ of Os10g0456800 was close to 0.75 (Figure 5e,g), suggesting that these candidate genes led to the differentiation of *indica* and *japonica*. In conclusion, the three promising candidate genes may be unique in the common wild rice (CLCWR, *O. rufipogon*).

## 3. Discussion

### 3.1. Accuracy of Phenotypic Identification

There are many methods for statistical analysis of callus browning, such as weighing the calli fresh [2], CBR and CBI [3,5], physiological and biochemical index measurement [27], and metabolite profiling and screening [4], which indicate that callus browning is affected by many factors. However, CBR and CBI are the most direct evaluation indexes. Compared to CBR, the CBI not only represents browning frequency and extent but also eliminates the error caused by personal factors [3,5]. In this study, to ensure the accuracy of the experiment, two indexes of CBR and CBI were used to evaluate the degree of callus browning(Figure 1), and there was a significant correlation between CBR and CBI (Table 2). At the same time, in order to minimize the error caused by person, phenotype observation is completed by one person, so as to ensure that the evaluation criteria of phenotype remain consistent. Single-point analysis and interval mapping showed that CBI could more accurately indicate the real existence of QTL (Table 3 and Table 4). Furthermore, investigation of the callus browning phenotype is not limited by season and region, and the method of culturing callus without bud removal is adopted at present, which saves time and reduces workload. In this study, we directly observed the phenotype on induction medium without de-budding for 30 days (Figure 1a–f), and there were significant differences between the introgression lines (Table 1), which ensures that the phenotype identification is done efficiently.

### 3.2. QTLs Identification of Callus Browning

The QTLs regulating callus browning have been located on chromosomes 1–12 using introgression lines between *indica* and *japonica* accessions [10,13,15,18] and introgression lines derived from crosses between *indica* rice and common wild rice accessions, such as Yuanjiang and Dongxiang [3,5,17]. In particular, QTLs related to callus browning have been repeatedly detected on chromosome 4. We also identified QTLs associated with lower CBR and CBI values mapping to chromosome 4, indicating that this region carries a main-effect quantitative locus acting as a hotspot for QTLs involved in callus browning. In previous studies, we first carried out map-based cloning of the QTL *qCBT3* (*BOC1, BROWNING OF CALLUS1*) on chromosome 3, which reduced callus browning and improved genetic transformation efficiency, of Yuanjiang common wild rice (Figure S1f) [3]. In the current study, we used interval mapping and identified QTLs associated with CBI on chromosomes 7 and 10. *qCBT10* was consistent with a previously identified QTL mapping near primer RM467 that was associated with callus subculture capability in the *indica* rice culture system using CSSLs between Zhenshan 97B and Nipponbare [18]. *qCBT7* has not yet been reported, indicating that it may be unique to CLCWR. It can also be seen that both the same and specific QTLs can be located in the introgression lines constructed by different *indica* rice varieties and different common wild rice varieties.

### 3.3. Integrating Linkage Analysis and WGCNA to Identify Key Genes for Callus Browning

Most studies on culturability have used QTL analysis as the sole source of gene identification, which is time consuming. As omics technologies have matured, researchers have begun combining the results of various omics analyses to quickly locate candidate genes. For example, a major QTL, *qCIR9.1*, for callus induction rate was located to a 100 kb region on chromosome 9 using 192 recombinant inbred lines between the *indica* accession YZX and the *japonica* accession 02428 and the use of bin map [10]. A single gene was obtained by combining DEG analysis [10]. In our study, combining QTL analysis, DEGs, and WGCNA, we identified three candidate genes in the *qCBT7* and *qCBT10* genomic intervals. *DCA1* encodes a transcriptional coactivator of *DST* expression to affect the expression of a peroxidase 24 precursor gene, whose encoding protein functions could eliminate H_2_O_2_. *DCA1*-overexpressing plants had less H_2_O_2_ than the control; however, *dca1* mutants had more H_2_O_2_ than plants from the corresponding wild type [36]. In our previous research, RNA-seq data combined with physiological and biochemical indicators suggested that *BOC1* may decrease cell senescence and cell death caused by oxidative stress to decrease the incidence of callus browning [3]. *DCA1* expression was higher in the introgression lines HCL183, HCL232, and YIL25, which have low callus browning values, than in the cultivars 9311 and TQ, which have severe callus browning phenotypes. In conclusion, we used QTL analysis combined with DEGs analysis and WGCNA of RNA-seq data to obtain candidate genes responsible for callus browning, which is an effective strategy.

### 3.4. Three Candidate Genes Have Potential Applications for Rice Biotechnology

In contrast to *indica* rice, *japonica* rice has better culturability potential. In our study, using publicly available data, we analyzed the *F*_ST_ of three candidate genes between the *indica* and *japonica* subspecies *O. sativa* and *O. rufipogon*. Significant differences showed that the three promising candidate genes were unique to the Chaling common wild rice, which might help improve the culture characteristics and genetic transformation efficiency of cultivated rice.

In the future, we will explore the functions of the three candidate genes, clarify their target genes, and study their molecular mechanisms and potential use value in influencing callus browning with the aim of diminishing or even inhibiting browning to improve *indica* rice genetic transformation efficiency for rice biotechnology.

## 4. Materials and Methods

### 4.1. Plant Materials

A cross between 9311, a high-yielding commercial *indica* rice (*Oryza sativa*) cultivar, and Chaling common wild rice (CLCWR, *O. rufipogon*) collected from Chaling county, Hunan, was used to generate introgression lines. A total of 124 rice introgression lines (BC_3_F_3_, named HCLs) were used to investigate browning.

### 4.2. Callus Induction and Proliferation Medium

NB medium (4072.3 mg/L salts) from Duchefa Biochemie B.V. (https://www.duchefa-biochemie.com/, accessed on 10 September 2021, Lot. No: P20416.01) was used as the basal medium, containing 2 mg/L of 2,4-D and 30 g/L sucrose. Then, it was adjusted to pH 5.8 and phytagel of 3 g/L for solidification according to our previous study [3].

### 4.3. Cultivation Process

Mature and dehulled seeds of 124 HCLs were placed in 50 mL triangle bottles and surface sterilized in 70% (*v*/*v*) ethanol for ~2 min. The seeds were then immersed in 15% (*w*/*v*) sodium hypochlorite solution and shaken continuously at 220 rpm for 15 min. Seeds were rinsed four times using sterile water, transferred to sterilized filter paper to air dry, and finally placed on a callus-induction medium. The dehulled seeds of each line were divided across three dishes (corresponding to three repetitions) containing 25 seeds each and placed at 28 °C in the dark for 30 days.

### 4.4. Phenotypic Identification and Analysis

The phenotypes were assessed as described in our previous study [3]. Tissue culture is not restricted by season and region, and its phenotype remains stable. We collected seeds from each introgression line and inoculated them for 30 days. Each introgression line material was inoculated in 3 Petri dishes (9 × 9) with approximately 25 seeds per dish. The callus browning was investigated after 30 days of dark culture. Due to the lack of seeds in some introgression lines or the contamination of the inoculation process, we finally investigated the callus browning of materials in 124 introgression lines. Browning phenotypes were divided into 5 grades: grade 0, browning observed on at most 10% of the calli produced (lightest browning); grade 1, 10% to 33% browning; grade 2, 33% to 66% browning; grade 3, 66% to 100% browning; grade 4, strong browning for all callus. The grades were calculated as follows:

(1) $$\mathrm{CBR}\;(\%)=\frac{number\;of\;calli\;showing\;browning}{total\;number\;of\;calli\;produced}\times 100;$$

(2) $$\mathrm{CBI}\;(\%)=\frac{sum\;of\;[number\;of\;calli\;at\;each\;browning\;level\;\times \;browning\;level]}{total\;number\;of\;calli\;produced\;\times \;highest\;browning\;level}$$

### 4.5. QTL Analysis

QTL analyses were performed with the SNP chip genotype data of 124 HCLs by the single-point method using Map Manager QTXb20 [38] and interval analysis using QTL IciMapping software V4.1 [39].

### 4.6. Transcriptome Analysis

RNA from 9311, HCL183, and HCL232 callus cultured for 30 days was extracted using an EASYspin Universal Plant RNA Kit with DNase (RN52, Aidlab, Beijing, China) and used for analysis. The raw sequencing data for the rice cultivar Teqing (TQ) and the introgression line YIL25 were obtained from our previous publication [3]. The gene count matrices were used as inputs to identify differentially expressed genes (DEGs) [40], with *P*_adj_  <  0.05 and abs(log2[fold change])  ≥  1. Jenn was used to generate a Venn diagram [41].

### 4.7. WGCNA

The R package WGCNA [42,43] was used to calculate the weighted association analysis. The parameters of the WGCNA were set as follows: the soft power was 10 (*R*^2^ = 0.8), mergeCutHeight was set as >0.75, and minModuleSize was set as >30.

### 4.8. qRT-PCR

To generate cDNA, 1 μg of RNA was used as a template for reverse transcription, while the oligo (dT)_18_ was employed for the synthesis of first-strand cDNA. The CFX96 real-time system (BioRad) was utilized for qPCR, where 5 ng of cDNAs, 4 μM of primers, and 5 μL of iQ SYBR Green Supermix (real-time PCR SYBR MIX, Bio-Rad, Hercules, CA, USA) were included in each reaction. To normalize gene expression data using the relative quantification method (2^–ΔΔCT^) [44], the rice housekeeping gene *Actin* (LOC_Os03g50885) was employed as an internal control. Please refer to Table S1 for the list of primers used in this analysis.

### 4.9. Statistical Analysis

The phenotyping data of CBR and CBI were calculated in Microsoft Excel. Then, we conducted data analysis by SPSS v25.0 (SPSS Inc., Chicago, IL, USA), including frequency distributions, variance (ANOVAs), correlation analyses of the two indices, and comparisons by two-tailed Student’s *t*-test.

## 5. Conclusions

To identify the QTLs associated with callus browning, CBR and CBI of 124 lines from Chaling common wild rice (*Oryza rufipogon* G.) with 9311 genetic backgrounds were evaluated. Combining 2059 SNPs, the two major QTLs, *qCBT7* on chromosome 7 and *qCBT10* on chromosome 10, were detected and the alleles from CLCWR reduced callus browning. RNA-seq data were analyzed, including DEGs and WGCNA, using light-browning introgression lines HCL183, HCL232, and YIL25 and heavy-browning *indica* cultivar 9311 and TQ. Three promising candidate genes (Os07g0620700, Os10g0361000, and Os10g0456800) were identified with upregulation, and qRT-PCR analysis of them showed the same results as RNA-seq. Meanwhile, these three candidate genes were obviously differentiated between the *indica* and *japonica* rice subspecies, *O. sativa* and *O. rufipogon*. Our findings not only provide an important gene target for improving culturability and genetic transformation efficiency but also a molecular framework that offers in-depth insight into callus browning.

## Figures and Tables

**Table 1:** ANOVA for CBR and CBI in HCLs.

Traits	Source of Variation	SS	df	MS	*F*	*p*-Value
CBR	Replication	0.456	90	0.005		
	total	4.623	212			
	line	4.167	122	0.034	6.746 **	1.31 × 10^−18^
CBI	Replication	0.592	90	0.007		
	total	7.907	212			
	line	7.315	122	0.06	9.122 **	2.405 × 10^−23^

** indicates significantly different at 0.01 probability level.

**Table 2:** Correlations between CBR and CBI.

	CBR	CBI
CBR	1	
CBI	0.792 **	1

** indicates significantly different at 0.01 probability level.

**Table 3:** QTL analysis of CBR and CBI in HCLs using the single-point method.

Trait	Chr. ^a^	Locus ^b^	QTL	PV(%) ^c^	*P* ^d^	Add ^e^
CBR	4	10,720,051	*qBR4-1*	10	0.00031	−5.35
	7	24,345,190	*qBR7-1*	10	0.00038	−5.36
	7	24,345,332	*qBR7-2*	8	0.00101	−9.67
	9	16,692,442	*qBR9-1*	16	0.00001	−6.94
	9	12,618,887	*qBR9-2*	13	0.00003	−5.92
	9	15,132,139	*qBR9-3*	13	0.00002	−6.22
	9	16,504,549	*qBR9-4*	12	0.00009	−9.51
	9	97,807,851	*qBR9-5*	9	0.00087	−5.49
	9	10,486,633	*qBR9-6*	9	0.00076	−7.53
	9	18,382,296	*qBR9-7*	9	0.00057	−5.13
	9	17,104,635	*qBR9-8*	8	0.00105	−5.51
	9	97,806,991	*qBR9-9*	8	0.00143	−7
	10	14,087,592	*qBR10-1*	10	0.00037	−5.47
	10	13,528,182	*qBR10-2*	9	0.00045	−5.52
CBI	2	46,187,291	*qBI2-1*	9	0.00071	−0.08
	4	10,720,051	*qBI4-1*	11	0.00014	−0.08
	7	24,345,190	*qBI7-1*	13	0.00003	−0.08
	7	24,345,332	*qBI7-2*	11	0.00017	−0.15
	7	88,806,091	*qBI7-3*	10	0.00038	−0.07
	7	77,390,231	*qBI7-4*	9	0.00072	−0.07
	7	12,434,577	*qBI7-5*	9	0.00054	−0.07
	7	15,404,459	*qBI7-6*	8	0.00179	−0.06
	7	18,251,259	*qBI7-7*	8	0.00134	−0.1
	7	22,320,372	*qBI7-8*	8	0.0017	−0.1
	7	24,690,967	*qBI7-9*	8	0.0011	−0.09
	9	15,132,139	*qBI9-1*	9	0.00048	−0.07
	9	16,692,442	*qBI9-2*	9	0.00061	−0.07
	9	12,618,887	*qBI9-3*	8	0.00155	−0.06
	10	14,087,592	*qBI10-1*	15	0.00001	−0.09
	10	13,528,182	*qBI10-2*	13	0.00003	−0.09
	10	17,073,302	*qBI10-3*	10	0.00029	−0.08
	10	13,727,931	*qBI10-4*	9	0.00079	−0.12

^a^: Chromosome; ^b^: physical location; ^c^: phenotypic variance; ^d^: the probability that the marker genotype did not affect the trait; ^e^: additive effect of allele from CLCWR.

**Table 4:** QTL analysis of CBR and CBI in HCLs using interval mapping.

Trait	Chr.	Left Marker	Right Marker	LOD	PV(%)	Add
CBR	1	38,384,198	39,169,324	4.1653	1.5038	−0.4482
	2	3,991,537	5,258,744	6.8527	1.6944	−15.3833
	2	6,478,167	7,064,337	4.2684	1.4617	−0.2163
	3	1,305,123	2,984,348	4.0297	1.582	−0.9482
	4	20,594,577	35,126,298	9.778	1.684	−0.2971
	5	3,443,919	5,376,334	6.4935	1.6121	−0.1325
	6	10,080,199	18,211,679	17.4229	2.9635	−19.9732
	6	18,211,679	20,978,032	2.7674	1.5154	−1.7639
	7	1,569,714	2,042,878	2.5546	1.3865	−0.6964
	7	2,667,022	4,012,634	3.3743	1.4399	−1.782
	7	6,813,655	7,739,024	7.2528	1.6284	−1.1897
	7	7,739,071	8,880,501	4.2682	1.472	−2.5481
	7	24,092,079	25,681,731	2.7068	1.547	−12.9071
	7	26,562,840	28,917,404	6.0147	1.6942	−14.8156
	8	15,795,968	19,682,615	8.5256	1.6275	−0.9063
	8	19,682,615	21,333,828	6.8575	1.6091	−1.0081
	8	24,878,660	26,386,382	6.3479	1.5513	−3.6428
	9	8,714,345	15,691,090	6.6571	0.9438	−0.2239
	10	10,588,708	17,676,090	9.2205	2.2374	−17.1459
	11	2,496,055	4,037,176	7.016	1.8231	−15.5316
	12	2,398,611	2,967,494	5.6647	1.601	−0.6781
CBI	7	24,092,079	25,681,731	3.9664	4.5413	−0.134
	10	10,588,708	17,676,090	3.0214	10.1809	−0.154

## Data Availability

The datasets generated during and/or analyzed during the current study are available from the corresponding author upon reasonable request.

## Supplementary Materials

The following supporting information can be downloaded at: https://www.mdpi.com/article/10.3390/genes14122138/s1, Figure S1: The callus browning phenotypes of TQ, YJCWR, and YIL25; Figure S2: Coexpression modules identified by WGCNA; Table S1: The primers for qRT-PCR.
