# Supplementary material for: Genetic and Transcriptome Analyses of Callus Browning in Chaling Common Wild Rice (Oryza rufipogon Griff.)
Source: Genes (Basel). 2023 Nov 27;14(12):2138. doi: 10.3390/genes14122138 (PMC10742633; doi:10.3390/genes14122138)
Supplement: Supplementary file 1 [file genes-14-02138-s001.zip › Supplementary Materials-1.pdf]

Supplementary Figure

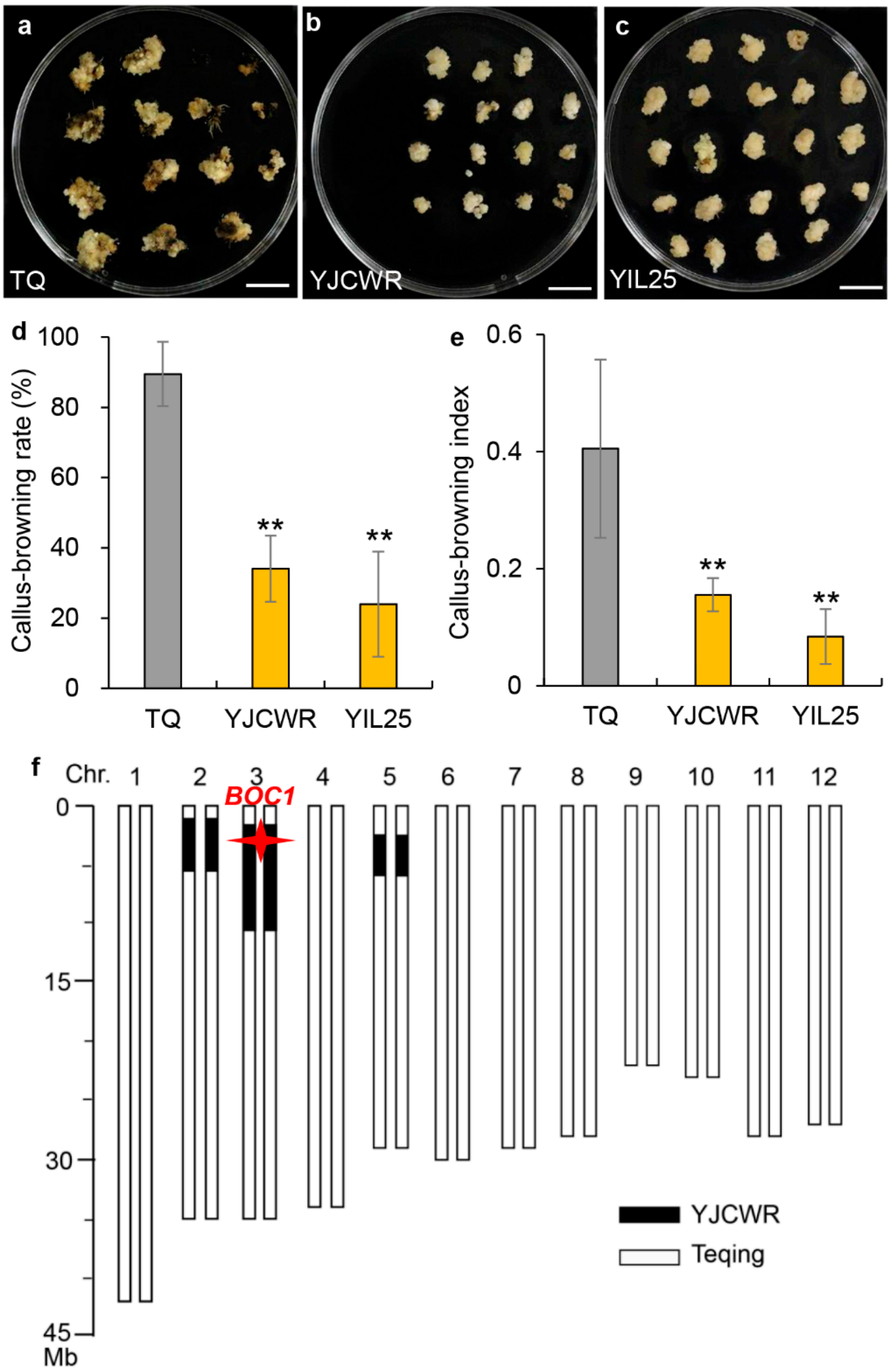

Figure S1. The callus browning phenotypes of TQ, YJCWR, and YIL25. **(a-b)** The comparison of callus browning in TQ, YJCWR, and YIL25. These mature seeds were inoculating on NB1 medium for 7 days and then transferred the resulting scutellum-derived calli to NB1 medium for 3 weeks of subculture (Zhang et al., 2020). **(d-e)** The comparison of CBR and CBI between TQ, YJCWR, and YIL25. **(f)** Graphic depiction of genotype of the introgression line YIL25 and *BOC1* was fine-mapped on the short arm of chromosome 3. The red five-pointed star indicated the location of *BOC1*.

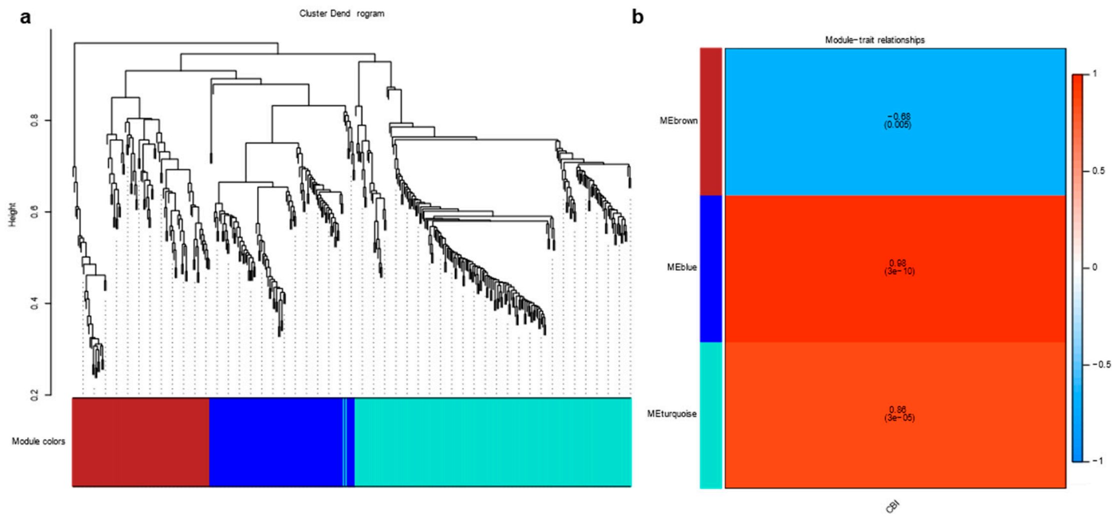

Figure S2. Coexpression modules identified by WGCNA. **(a)** The dendrogram was obtained by clustering dissimilarities based on consistent topological overlap, with the corresponding module colors indicated by color rows. **(b)** The table is color-coded to show correlations according to the corresponding color legend. Red indicates a positive correlation, while green indicates a negative correlation. The numbers in the boxes represent the correlations between module stages and signature genes. The corresponding *p*-values are shown in parentheses below.

Supplementary Table

Table S1 The primers for qRT-PCR

Primer name	Forward primer (5'−3')	Reverse primer (5'−3')
Os03g0718100 (*actin*)	TGGCATCTCTCAGCACATTCC	TGCACAATGGATGGGCCAGA
Os07g0620700	ACCTCGATATCGGCCTAAAC	GTGAAGGCTCTCGTGATTTG
Os10g0361000	TAGCTCCATCGACCAGTGTT	TGTGCCCCACACATAGTACA
Os10g0456800	AATGCGGGTGTTGCTACAGT	ATGCGCTCTCATCACGTTCA
